# *Lactobacillus rhamnosus* and *Lactobacillus gasseri* ameliorate alcohol-induced liver injury and gut microbiota dysbiosis in rats

**DOI:** 10.3389/fnut.2026.1887275

**Published:** 2026-10-02

**Authors:** Yi Zhang, Haiting Liu, Yao Fu, Nan Cai, Yawen An, Hanqing Liu, Xiaojian Jia, Dajun Zhang

**Affiliations:** 1 Shenzhen Kangning Hospital & Shenzhen Mental Health Center, Shenzhen, China; 2 Clinical College of Mental Health, Shenzhen University Health Science Center, Shenzhen, China; 3 Affiliated Mental Health Center, Southern University of Science and Technology, Shenzhen, China; 4 School of Pharmacy, Shenyang Medical College, Shenyang, China; 5 Research & Teaching Department, The Fourth People's Hospital of Shenzhen (Shenzhen Samii Medical Center), Shenzhen, China; 6 Review and Evaluation Department, The Fourth People's Hospital of Shenzhen (Shenzhen Samii Medical Center), Shenzhen, China

**Keywords:** alcohol-induced liver injury, gut microbiota, *Lactobacillus gasseri*, *Lactobacillus rhamnosus*, probiotics

## Abstract

**Introduction/objective:**

This study aimed to evaluate whether *Lactobacillus rhamnosus* and *Lactobacillus gasseri* could alleviate alcohol-induced liver damage, behavioral changes, and gut microbiota dysbiosis in rats. And sought to better understand the role of Lactobacillus-mediated microbiota modulation in alcohol-induced liver damage.

**Methods:**

Male SD rats were first subjected to 1 week of ethanol adaptation, followed by 4 weeks of feeding with a Lindros alcohol liquid diet containing 6.2% ethanol to establish the ALD model. Subsequently, the rats received 2 weeks of either *L. rhamnosus* or *L. gasseri* (1 × 10^9^ CFU/rat/day) treatment while continuing to receive the Lindros alcohol liquid diet containing 6.2% ethanol. The control group were treated with PBS. Serum fibroblast growth factor 21 (FGF21), lipopolysaccharide (LPS), alanine aminotransferase (ALT), and aspartate aminotransferase (AST) were determined, along with hepatic TNF-α expression. Behavioral performance was assessed using the open-field test, and gut microbiota composition was analyzed by 16S rRNA gene sequencing.

**Results:**

Alcohol-fed rats showed significantly increases in serum FGF21, LPS, ALT, AST, and hepatic TNF-α levels, accompanied by reduced locomotor activity and gut microbial diversity. Supplementation with either *L. rhamnosus* or *L. gasseri* mitigated these changes. Notably, *Lactobacillus gasseri* demonstrated a more pronounced protective effect on liver injury markers. *Lactobacillus* treatment partially restored microbial richness, increased beneficial genera such as *Monoglobus*, and reduced alcohol-associated taxa including *Bacteroides* and *Alloprevotella*. Functional prediction analysis indicated significant alterations in pathways related to sulfur metabolism, purine metabolism, the sulfur cycle, and butanoate metabolism.

**Discussion:**

Both *L. rhamnosus* and *L. gasseri* effectively attenuated alcohol-induced liver injury, inflammation, and behavioral impairments in rats. These effects are likely through modulation of gut microbiota composition and function.

**Conclusion:**

Our work suggest that probiotic intervention may represent a promising therapeutic strategy for the management of alcohol-induced disorders.

## Graphical abstract

**Figure:**
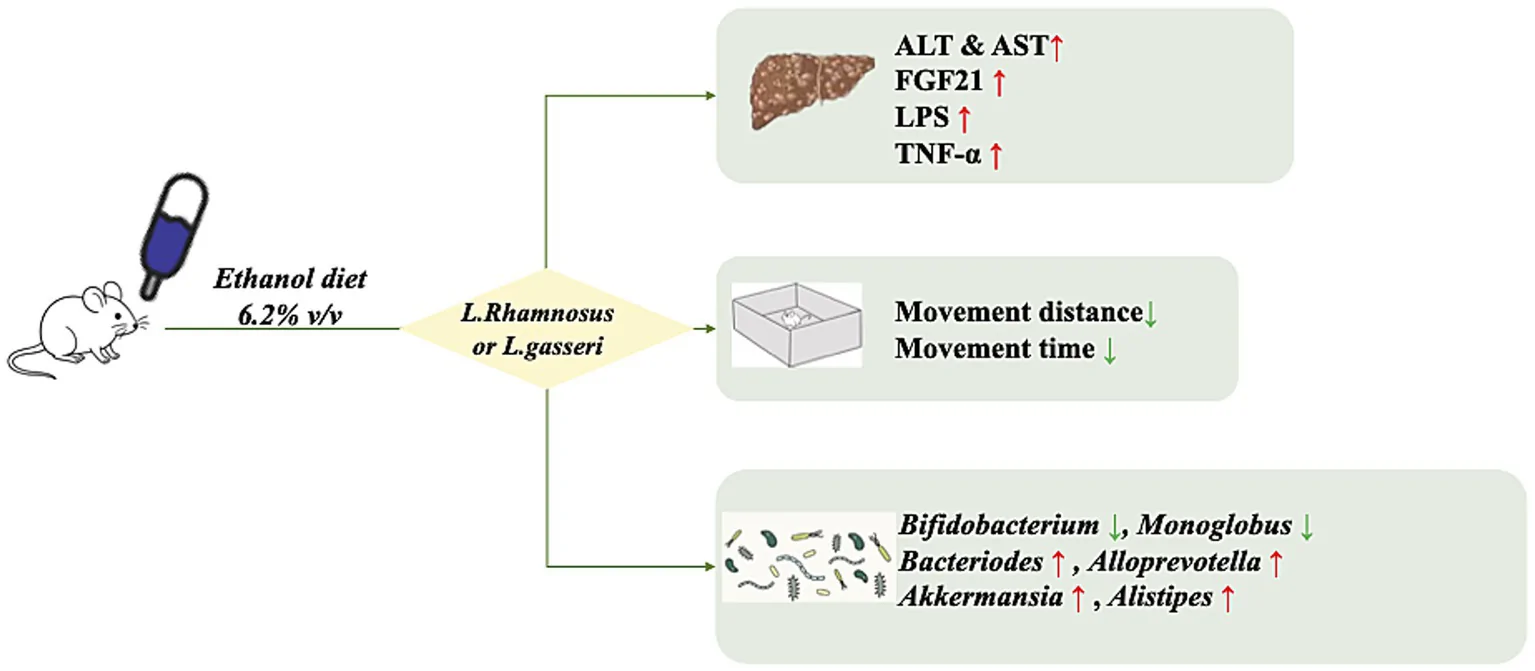

## Introduction

Alcohol abuse is a major public health problem, with inappropriate use of alcohol contributing to 2.6 million deaths, millions of disabilities, and ill health globally each year, placing a significant burden on individuals, families, and society (1). According to the 2024 Global Status Report on Alcohol and Health published by the World Health Organization (WHO), alcohol-related diseases pose a serious risk to human health and public safety (1). Among the Chinese, a meta-analysis of studies published before January 2014 found that the prevalence of alcohol dependence was 2.2% for current cases and 3.7% over a lifetime, which are comparable to rates reported in Western countries (2).

Recent investigations suggest that imbalances in gut microbiota may contribute to alcohol related diseases, including alcohol dependence and alcohol-associated liver disease (3–5). Evidence from rodent and non-human primate experiments, as well as clinical observations in individuals with alcohol use disorder, shows that prolonged alcohol intake can alter gut microbial composition and trigger dysbiosis (6, 7). The dysbiosis result in a shift in microbial composition characterized by reduced diversity, which is often accompanied by reductions in several commensal bacteria, including *Lactobacillus*, *Faecalibacterium*, and, in some studies, *Akkermansia*, although changes in *Akkermansia* have been reported to vary across different experimental models and disease stages, and overgrowth of inflammation-associated taxa (e.g., *Proteobacteria* and Enterobacteriaceae) (8, 9).

A key consequence of alcohol-induced dysbiosis is increased intestinal permeability, often termed “leaky gut.” Alcohol and its metabolites, such as acetaldehyde, compromise tight junctions in the gut epithelium, allowing bacterial endotoxins like lipopolysaccharides (LPS) to translocate into the bloodstream. This endotoxemia activates immune pathways including Toll-like receptor 4 (TLR4), leading to the release of pro-inflammatory cytokines such as TNF-α, IL-6, IL-1β, and promoting a state of systemic inflammation. In AUD, this inflammatory response accelerates tissue injury, particularly in the liver, as seen in alcoholic liver disease. In addition, inflammation extends to the central nervous system, where it contributes to neuroinflammatory changes in brain regions associated with reward processing, stress regulation, and craving (10–12).

The gut-brain axis further amplifies these effects. Metabolites produced by the gut microbiota, including short-chain fatty acids (SCFAs), together with inflammatory mediators, influence key neurotransmitter systems such as serotonin and GABA, both of which are disrupted in AUD (13). For instance, reduced SCFA production due to dysbiosis weaken intestinal barrier integrity and diminish anti-inflammatory signaling. At the same time, shifts in tryptophan metabolism increases kynurenine pathway activity, which has been linked to increased anxiety, depressive symptoms, and relapse risk (13, 14). These processes appear to be mutually reinforcing, as alcohol-induced inflammation aggravates gut microbial imbalance, while microbiota alterations, in turn, sustain neurobehavioral features of addiction.

*Lactobacillus*-based probiotics offer promise for the management of alcohol related diseases. Strains like *Lactobacillus rhamnosus* GG (LGG), *Lactobacillus plantarum*, *Lactobacillus reuteri*, and *Lactobacillus casei* restore microbial diversity, enhance gut barrier function, suppress inflammation, and reduce oxidative stress by scavenging reactive oxygen species and upregulating antioxidants like superoxide dismutase (9, 15). Clinical evidence indicates that 6-month LGG treatment in patients with moderate alcohol-associated hepatitis significantly lowers heavy drinking to social or abstinent levels (e.g., <4 drinks/week), improves liver biomarkers, and modulates the gut-brain axis to curb cravings (16). Preclinical studies further support LGG’s role in elevating fibroblast growth factor 21 (FGF21) to regulate dopamine signaling and GABA production, potentially alleviating dependence (17).

However, the mechanism by which lactobacillus regulates gut microbiota homeostasis and thus affects the treatment effect of alcohol related diseases remains unclear. In this study, we chose two commonly used *lactobacillus* probiotic strains *L. rhamnosus* and *Lactobacillus gasseri* to investigate the potential therapeutic effects of *L. rhamnosus* and *L. gasseri* and their effects on the gut microbiota.

## Methods

### Rat breeding and experimental design

Six-week-old specific-pathogen-free (SPF) male Sprague–Dawley (SD) rats (*n* = 24; average body weight: 150 g) were obtained from the Guangdong Medical Experimental Animal Center (License No. SYXK [Yue] 2021-0001, Guangzhou, China). Only male rats were used in this study to reduce biological variability associated with sex hormones and the estrous cycle and to maintain consistency with established rodent models of alcohol-associated liver disease. The animals were housed under controlled environmental conditions (temperature: 21 ± 2 °C; humidity: 50% ± 5%) with a 12 h light/dark cycle and provided with standard chow and tap water ad libitum. All experimental procedures were approved by the Animal Care and Use Committee of the Shenzhen Peking University–Hong Kong University of Science and Technology Medical Center, China (Approval No. 2024–541).

After 7 days of acclimatization, the rats were randomly assigned to four groups (d-7, *n* = 6 per group): a control group (CG), an alcohol-fed group (Alc), a *L. rhamnosus-*treated group (LGG), and a *L. gasseri*-treated group (Ge). The CG group received a standard liquid diet for 7 weeks. The Alc group received a liquid diet containing ethanol, SD rats were first subjected to 1 week of ethanol adaptation, with the ethanol concentration gradually increased from 0 to 6.2% (v/v), followed by 4 weeks of feeding with a Lindros alcohol liquid diet containing 6.2% ethanol to establish the ALD model. Subsequently, the rats received 2 weeks of either *L. rhamnosus* (LGG) or *L. gasseri* (Ge) (1 × 10^9^ CFU/rat/day) treatment while continuing to receive the Lindros alcohol liquid diet containing 6.2% ethanol. The control group (CG) were treated with PBS in the last 2 weeks while continuing to receive the Lindros alcohol liquid diet containing 6.2% ethanol (Figure 1A).

**Figure 1:**
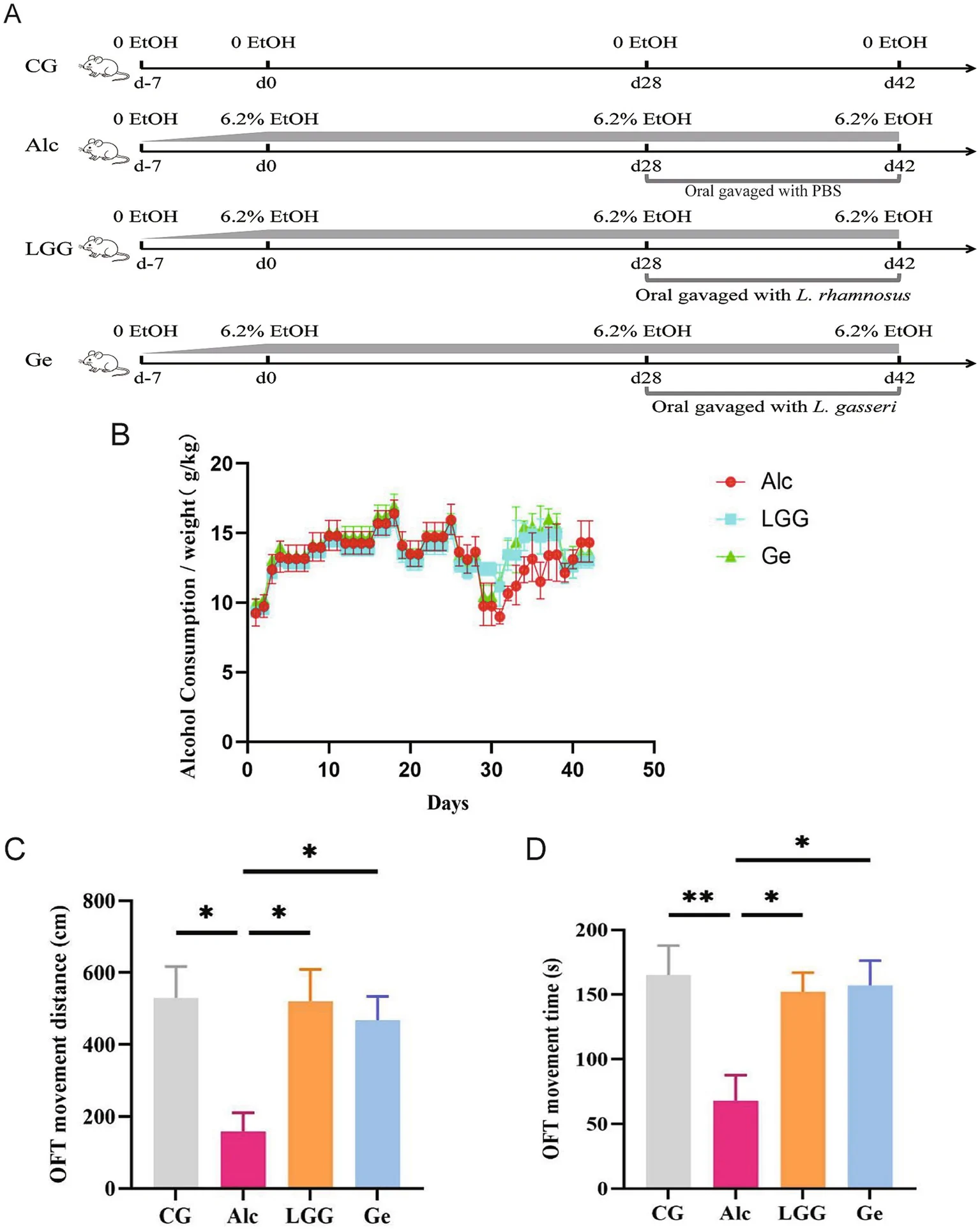
Experimental design, alcohol consumption, and behavioral changes in each group (*n* = 6 per group). **(A)** Schematic representation of the experimental procedures for alcohol feeding and *Lactobacillus* treatment in each group. **(B)** Alcohol consumption in the Alc, LGG, and Ge groups during the experiment. **(C,D)** Open-field test (OFT) results showing movement distance and duration in each group. *p < 0.05, **p < 0.01.

*Lactobacillus rhamnosus* and *L. gasseri* was commercially obtained from Shandong Zhongke Jiayi Bioengineering Co., Ltd. (Shandong, China). The strains were routinely cultured anaerobically in de Man–Rogosa–Sharpe (MRS) broth at 37 °C before administration. Fresh bacterial cultures were prepared on the day of gavage, and the bacterial suspension was adjusted to a final concentration of 1 × 10^9^ CFU/mL based on the optical density at 600 nm (OD600).

Rats in the alcohol-fed groups (Alc, LGG, Ge) were fed a Lindros alcohol liquid diet (catalog No. TP 4060E, TROPHIC Animal Feed High-Tech Co. Ltd., China), a high-fat, low-carbohydrate modification of the standard Lindros alcohol liquid diet. Which has been widely used to establish experimental alcohol-associated liver disease (18, 19). The diet provided 16% of total calories from protein, 44% from fat, 5.5% from carbohydrate, and 34.5% from ethanol. During the adaptation period, the ethanol concentration was gradually increased to final concentration 6.2% (v/v) and was then maintained throughout the subsequent experimental period. Rats in the control group (CG) were pair-fed an isocaloric liquid diet without ethanol (catalog No. TP 4060EC, TROPHIC Animal Feed High-Tech Co. Ltd., China), in which the calories derived from ethanol were replaced by carbohydrates to ensure equivalent caloric intake between groups.

To maintain consistency between groups, caloric intake was controlled across the entire experimental period. Ethanol intake was measured daily, and body weight was recorded weekly. Fecal samples were collected at the end of the experiment. And behavioral tests were then conducted. Following behavioral tests, rats were deeply anesthetized with isoflurane until loss of pedal reflex. Blood was collected by cardiac puncture, followed by cervical dislocation to ensure death. The rats were then dissected to collect liver tissue. Blood samples were centrifuged at 3,000 rpm, 10 min at 4 °C to obtain serum. Liver tissues were flash-frozen in liquid nitrogen and stored at −80 °C for further analysis.

### Biochemical analyses

Enzyme-linked immunosorbent assay (ELISA) was used to measure serological parameters, including lipopolysaccharide (LPS), Alanine aminotransferase (ALT), aspartate aminotransferase (AST) and fibroblast growth factor 21 (FGF21), according to the manufacturers’ instructions. The ALT and AST ELISA kits were purchased from Medicalsystem Biotechnology Co., Ltd. (Ningbo, China) (ALT: catalog No. H001; AST: catalog No. H002). The LPS ELISA kit was obtained from Wuhan Saipei Biotechnology Co., Ltd. (Wuhan, China; catalog No. SP37177), and the FGF21 ELISA kit was purchased from Proteintech Group, Inc. (United States; catalog No. KE10095).

Hepatic levels of tumor necrosis factor-α (TNF-α) was quantified by Western blotting. Briefly, 100 mg liver samples were washed with phosphate-buffered saline (PBS), and homogenized in 1 mL ice-cold RIPA lysis buffer supplemented with protease inhibitors using a mechanical homogenizer. After which lysates were kept on ice for 30 min with vortexing for 30s every 10 min. Samples were then centrifugation at 12,000 × *g* for 15 min at 4 °C, and the clarified supernatant was collected for protein extraction. Total protein concentrations were measured using a bicinchoninic acid (BCA) assay. Equal amounts of protein were separated by 10% SDS–PAGE and transferred to polyvinylidene difluoride (PVDF) membranes. The membranes were blocked in Tris-buffered saline with 0.1% Tween-20 (TBST) containing 5% skim milk for 1 h at room temperature, then incubated overnight at 4 °C with the following primary antibodies: anti–TNF-α (rabbit polyclonal antibody, Affinity Biosciences; catalog No. AF7014) and anti–β-tubulin (mouse monoclonal antibody, TransGen Biotech Co., Ltd.; catalog No. HC101). Following three times TBST washes, membranes were incubated with HRP-linked secondary antibodies for 2 h at room temperature. Immunoreactive signals were detected using enhanced chemiluminescence and captured with a chemiluminescent imaging system.

### Open-field test (OFT)

The open-field test (OFT) was conducted between 8:30 a.m. and 12:30 p.m. Rats were individually tested in an open-field apparatus (100 cm × 100 cm × 40 cm; length × width × height) provided by Beijing Zhongshi Dichuang Science and Technology Development Co., Ltd. Each rat was gently placed in one corner of the chamber, and after a 1-min acclimation period, spontaneous activity was recorded for 5 min using a video tracking system (Labmaze V3.0, Zhongshi Technology). The total distance traveled was used as an index of locomotor activity, whereas the time spent and distance traveled in the central area (the inner 25% of the total surface) were used as indicators of anxiety-like behavior.

### 16S rDNA sequencing and analysis

According to the original study design, four biological replicates from each group were included for 16S rRNA gene sequencing and subsequent microbiome analysis. No predefined random sampling strategy was implemented for the selection of these samples. Importantly, sample selection was not based on alcohol consumption, behavioral performance, or other experimental outcomes, and the same sampling approach was applied across all experimental groups.

Microbial DNA was extracted from freshly collected rat fecal samples using a commercial stool DNA extraction kit, and 16S rRNA gene sequencing was performed by Novogene (Beijing, China). Briefly, the V4 hypervariable region of the 16S rRNA gene was amplified using the universal primers 515F–806R. PCR products were purified using magnetic bead purification, and quantified libraries were pooled and sequenced on the Illumina NovaSeq 6,000 platform. Paired-end reads were merged using FLASH software (version 1.2.11; http://ccb.jhu.edu/software/FLASH/) (20), and low-quality reads were filtered using fastp (version 0.23.1) (21) to obtain high-quality clean tags. Species annotation and downstream data analyses were performed using QIIME2 software.

### Statistical analysis

All statistical analyses were performed using SPSS software (version 27.0; IBM, United States), and figures were generated using GraphPad Prism (version 9.0; GraphPad Software, United States). Data are presented as mean ± standard deviation (SD). For normally distributed data, one-way analysis of variance (ANOVA) was used to compare differences among groups. A *p*-value < 0.05 was considered statistically significant.

For microbiota analysis, alpha diversity was assessed using the indices Observed_OTUs, Chao1, Shannon, Simpson, Dominance, Good’s coverage, and Pielou_e. Beta diversity was calculated based on weighted and unweighted UniFrac distances at the ASV level in QIIME2. To evaluate community structure differentiation, a series of statistical analyses—including ANOSIM, ADONIS, multi-response permutation procedure (MRPP), SIMPER, *t*-test, MetagenomeSeq, and LEfSe—were conducted. Western blot band intensities were quantified by ImageJ software (version 1.8.0; NIH, United States).

## Results

### Changes in alcohol consumption and behaviors in alcohol-fed and *Lactobacillus*-treated rats

In this experiment, rats in all three alcohol-exposed groups (Alc, LGG, and Ge) consumed more than 8 g/kg/day of ethanol (calculated as alcohol intake per body weight over 24 h; Figure 1B). This level of intake has previously been shown to induce gut barrier disruption and inflammatory liver injury in rats (22).

Consistent with this exposure, chronic alcohol consumption led to a reduction in spontaneous locomotor activity. Compared with CG group, Alc group rats exhibited a shorter total movement distance (*p* < 0.05) and decreased movement time (*p* < 0.01) (Figures 1C,D). In contrast, rats in LGG or Ge group showed significant improvement in both parameters (*p* < 0.05) compared with Alc group. Together, these findings suggest that prolonged alcohol intake impairs locomotor behavior in rats, whereas *Lactobacillus* supplementation partially reverses these deficits and helps restore normal motor activity.

### Changes in biochemical parameters in alcohol-fed and *Lactobacillus*-treated rats

Compared with the CG group, serum levels of fibroblast growth factor 21 (FGF21) and lipopolysaccharide (LPS) were significantly elevated in Alc group rats (*p* < 0.01) (Figures 2A,B). Similarly, serum alanine aminotransferase (ALT) and aspartate aminotransferase (AST) levels were markedly increased in the alcohol-fed group (ALT, *p* < 0.001; AST, *p* < 0.05) (Figures 2C,D). In addition, hepatic tumor necrosis factor-α (TNF-α) levels were significantly higher in Alc group than in CG group (*p* < 0.001) (Figures 2E,F), indicating alcohol-induced hepatic inflammation and injury.

**Figure 2:**
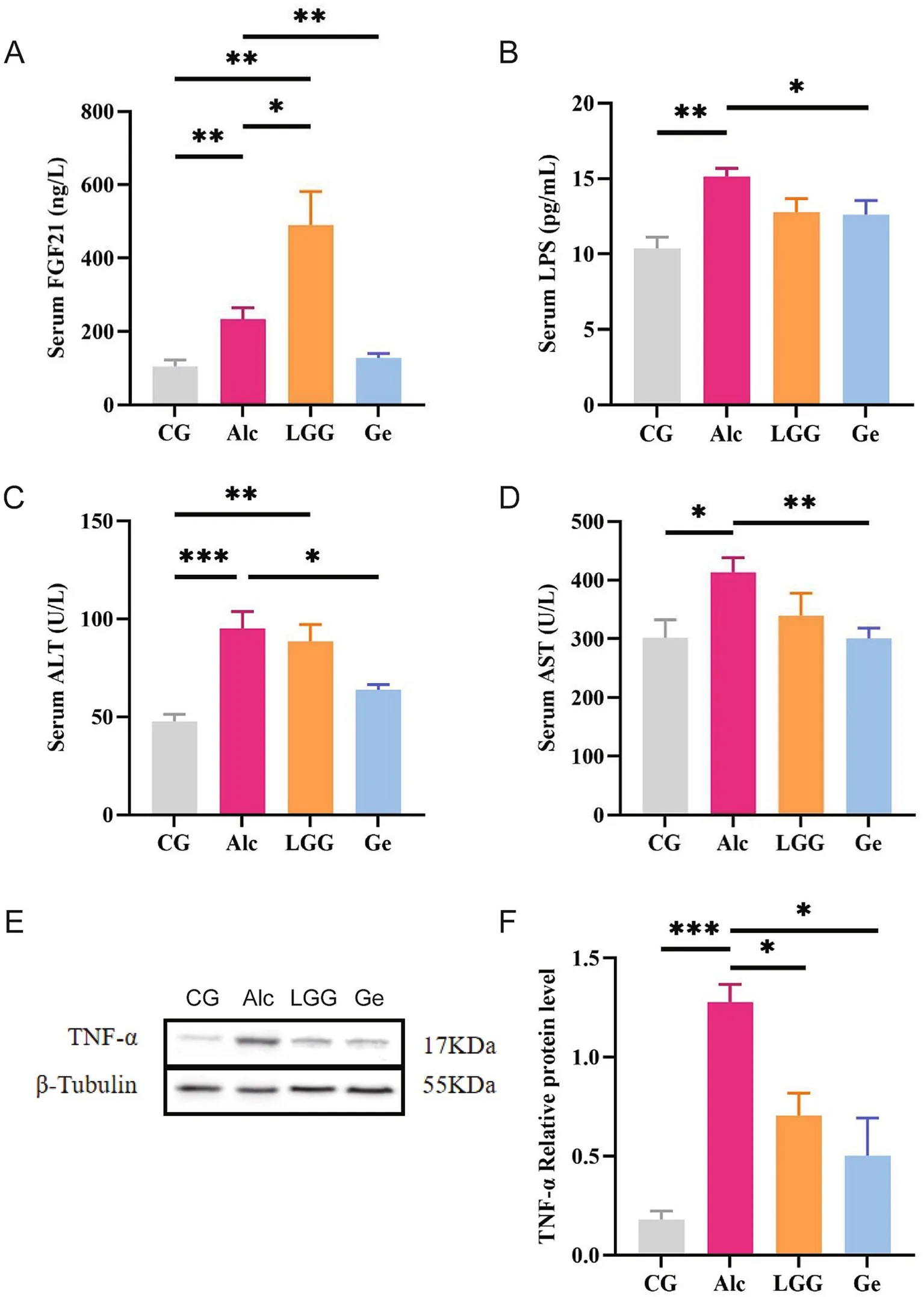
Changes in biochemical parameters in alcohol-fed and *Lactobacillus*-treated rats. **(A–D)** Serum levels of FGF21 **(A)**, LPS **(B)**, ALT **(C)**, and AST **(D)** in each group, *n* = 6 per group. **(E,F)** Western blot images **(E)** and quantitative analysis **(F)** of hepatic TNF-α expression, *n* = 3 per group. FGF21, fibroblast growth factor 21; LPS, lipopolysaccharide; ALT, alanine aminotransferase; AST, aspartate aminotransferase; TNF-α, tumor necrosis factor-α. *p < 0.05, **p < 0.01, ***p < 0.001.

Compared with the Alc group, serum FGF21 levels were significantly higher in the *LGG* group (*p* < 0.05), whereas they were significantly lower in the *Ge* group (*p* < 0.01) (Figure 2A). Additionally, serum LPS levels decreased by approximately 16% in the LGG group and were significantly reduced in the Ge group (*p* < 0.05) compared with alcohol-fed rats (Figure 2B).

In the LGG group, serum ALT and AST levels decreased by approximately 7 and 18%, respectively, relative to the alcohol-fed group, although these reductions did not reach statistical significance. In contrast, *L. gasseri* supplementation led to significant decreases in both serum ALT and AST levels compared with the alcohol-fed group (ALT, *p* < 0.05; AST, *p* < 0.01) (Figures 2C,D). Furthermore, hepatic TNF-α levels were significantly lower in both LGG and Ge group rats compared with Alc group rats (*p* < 0.05) (Figures 2E,F), suggesting that both probiotic strains exerted anti-inflammatory and hepatoprotective effects against alcohol-induced liver injury.

### Alterations in gut microbiota in alcohol-fed and *Lactobacillus*-treated rats

The richness indices (Chao and observed_features) and diversity indices (Shannon and Simpson) were used to evaluate the effects of alcohol consumption and *Lactobacillus* treatment on the gut microbiota. The richness indices were lower in the Alc group compared with the CG group, whereas both *L. rhamnosus* and *L. gasseri* treatments increased microbial richness. However, no significant differences were detected in the diversity indices among any of the groups (Figures 3A–D), indicating that alcohol intake and subsequent *Lactobacillus* treatment had little impact on overall gut microbial diversity.

**Figure 3:**
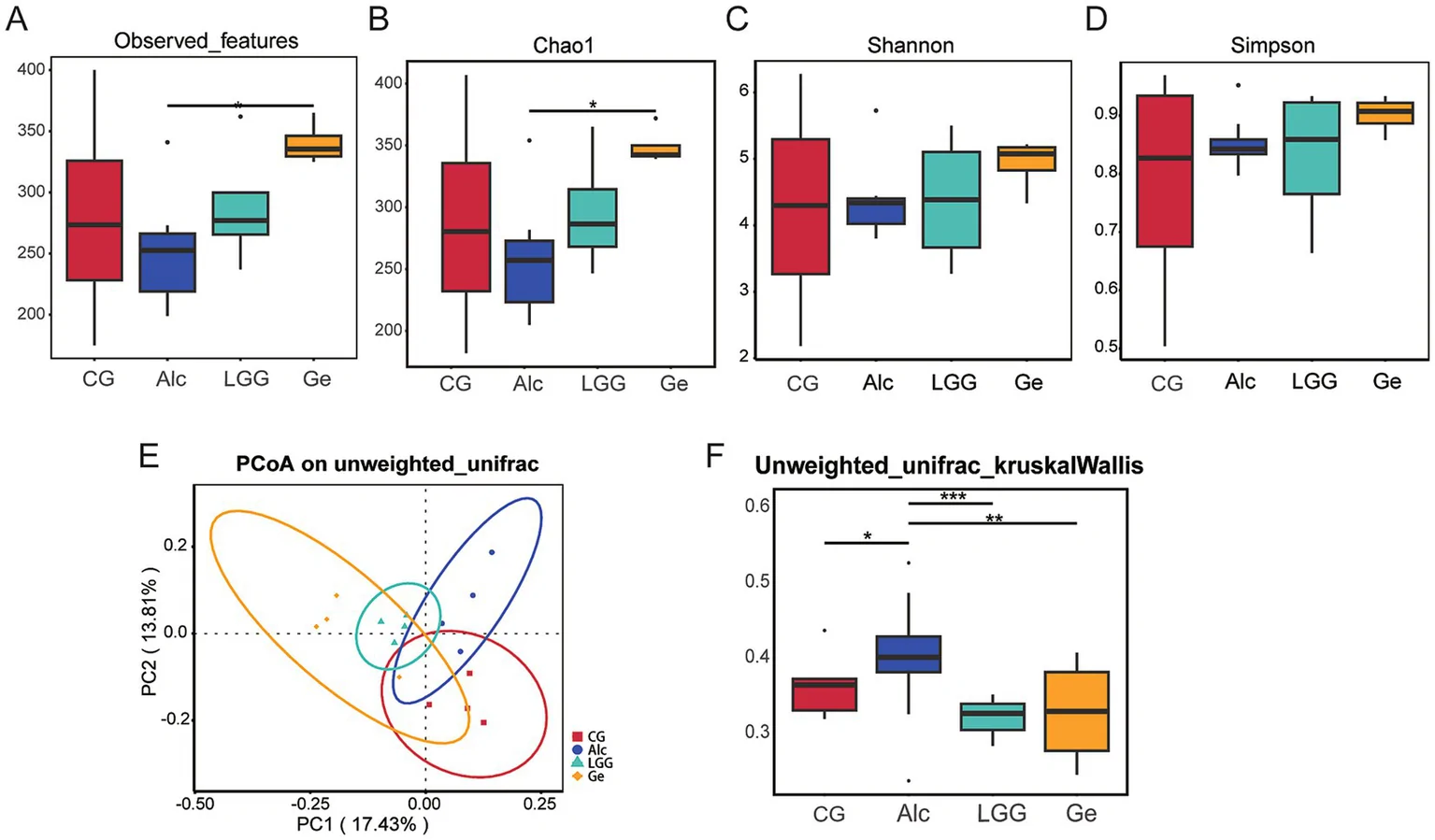
Changes in gut microbiota community structure among groups (*n* = 4 per group). **(A–D)** α-diversity analysis across groups: **(A)** *Observed_features*: reflects the number of distinct taxa detected within each community. **(B)** *Chao1 index*: an estimator of true species richness within a community. **(C)** *Shannon index*: a diversity measure accounting for both species richness and evenness. **(D)** *Simpson index*: reflects the probability that two randomly selected individuals from a sample belong to the same species, incorporating both richness and evenness. **(E)** Principal coordinates analysis (PCoA) plot based on unweighted_uniFrac distances among groups. **(F)** Kruskal_Wallis analysis based on unweighted_uniFrac values comparing microbial community structures between groups. *p < 0.05, **p < 0.0, ***p < 0.001.

Principal coordinate analysis (PCoA) using unweighted UniFrac distances showed clear separation of samples among the experimental groups, indicating marked differences in microbial community composition (Figure 3E). In particular, the gut microbiota of Alc group differed significantly from that of CG group (*p* < 0.05), as well as that from LGG (*p* < 0.001) and Ge group (*p* < 0.01) (Figure 3F).

To identify the bacterium most responsive to alcohol exposure and *Lactobacillus* intervention, we examined changes in relative abundance at both the phylum and genus levels. At the phylum level, the gut microbiota was dominated by four major groups: Firmicutes, Proteobacteria, Bacteroidetes, and Actinobacteria. Compared with CG group, alcohol intake was associated with a reduction in Firmicutes and a concomitant increase in Bacteroidetes and Verrucomicrobiota. *Lactobacillus* supplementation partially reversed these shifts. Specifically, the LGG group showed a significant reduction in Bacteroidetes abundance (*p* < 0.05), and the Ge group showed a significant decrease in Verrucomicrobiota abundance (*p* < 0.05) (Figure 4A and Supplementary Figure S1A). At the genus level, alcohol-fed rats exhibited significantly increased abundances of *Akkermansia*, *Alloprevotella*, *Bacteroides*, *Alistipes*, *Rikenellaceae_RC9_gut_group*, *Desulfovibrio*, and *Ruminococcus* (*p* < 0.05), whereas *Bifidobacterium* was significantly decreased (*p* < 0.05). In the *L. rhamnosus*–treated group, *Alloprevotella*, *Alistipes*, *GCA-900066575*, *Rikenellaceae_RC9_gut_group*, and *Prevotellaceae_UCG-001* were significantly reduced (*p* < 0.05), while *Monoglobus*, *Ligilactobacillus*, and *[Eubacterium]_siraeum_group* were significantly enriched (*p* < 0.05). In the *L. gasseri*–treated group, *Akkermansia*, *Alloprevotella*, and *Alistipes* were significantly decreased (*p* < 0.05), whereas *Colidextribacter*, *Christensenellaceae_R-7_group*, *Lachnospiraceae_NK4B4_group*, and *[Eubacterium]_siraeum_group* were significantly increased (*p* < 0.05) (Figure 4B and Supplementary Figure S1B).

**Figure 4:**
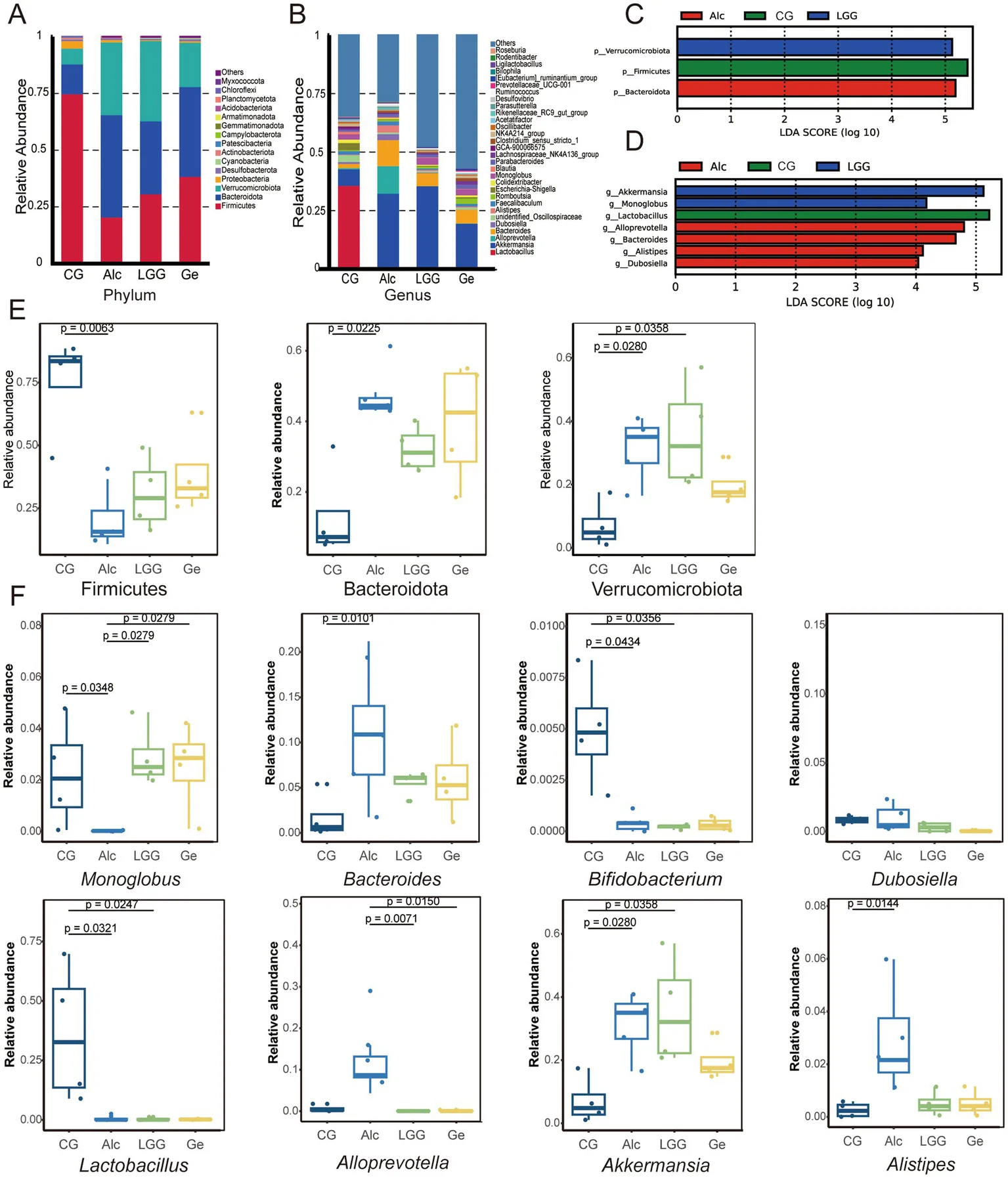
Changes in species abundance among groups (*n* = 4 per group). **(A,B)** Composition and relative abundance of microbial communities at the phylum **(A)** and genus **(B)** levels. **(C,D)** LEfSe analysis of microbial communities between groups, showing linear discriminant analysis (LDA) scores at the phylum **(C)** and genus **(D)** levels. **(E)** Distribution of microbial abundance for the three significantly different phyla. **(F)** Distribution of microbial abundance for the eight significantly different genera.

Linear discriminant analysis effect size (LEfSe) further identified characteristic taxa differentiating the groups (Figures 4C,D). At the genus level, *Lactobacillus* was markedly enriched in the control group, while *Alistipes*, *Alloprevotella*, *Bacteroides*, and *Dubosiella* were significantly increased in alcohol-fed rats. After *L. rhamnosus* treatment, *Akkermansia* and *Monoglobus* were notably enriched. These genera belong primarily to the phyla Firmicutes (*Lactobacillus*, *Dubosiella*, *Monoglobus*), Bacteroidetes (*Alistipes*, *Alloprevotella*, *Bacteroides*), and Verrucomicrobiota (*Akkermansia*) (Figure 4D). Figures 4E,F presents a box plot illustrating the distribution of microbial abundance at both the phylum and genus levels, confirming the significant upregulation or downregulation of these genera in alcohol-fed and *Lactobacillus*-treated rats.

### Functional alterations in alcohol-fed and *Lactobacillus*-treated rats

To better understand the functional relevance of the observed changes in microbial composition, we performed functional prediction using PICRUSt2 based on the Kyoto Encyclopedia of Genes and Genomes (KEGG) database. Relative abundances of predicted functions were compared across groups using clustering analysis to assess potential functional shifts. Venn diagram showing shared and group-specific KEGG Orthologs (KOs) were presented in Figure 5A, principal component analysis (PCA) results was shown in Figure 5B.

**Figure 5:**
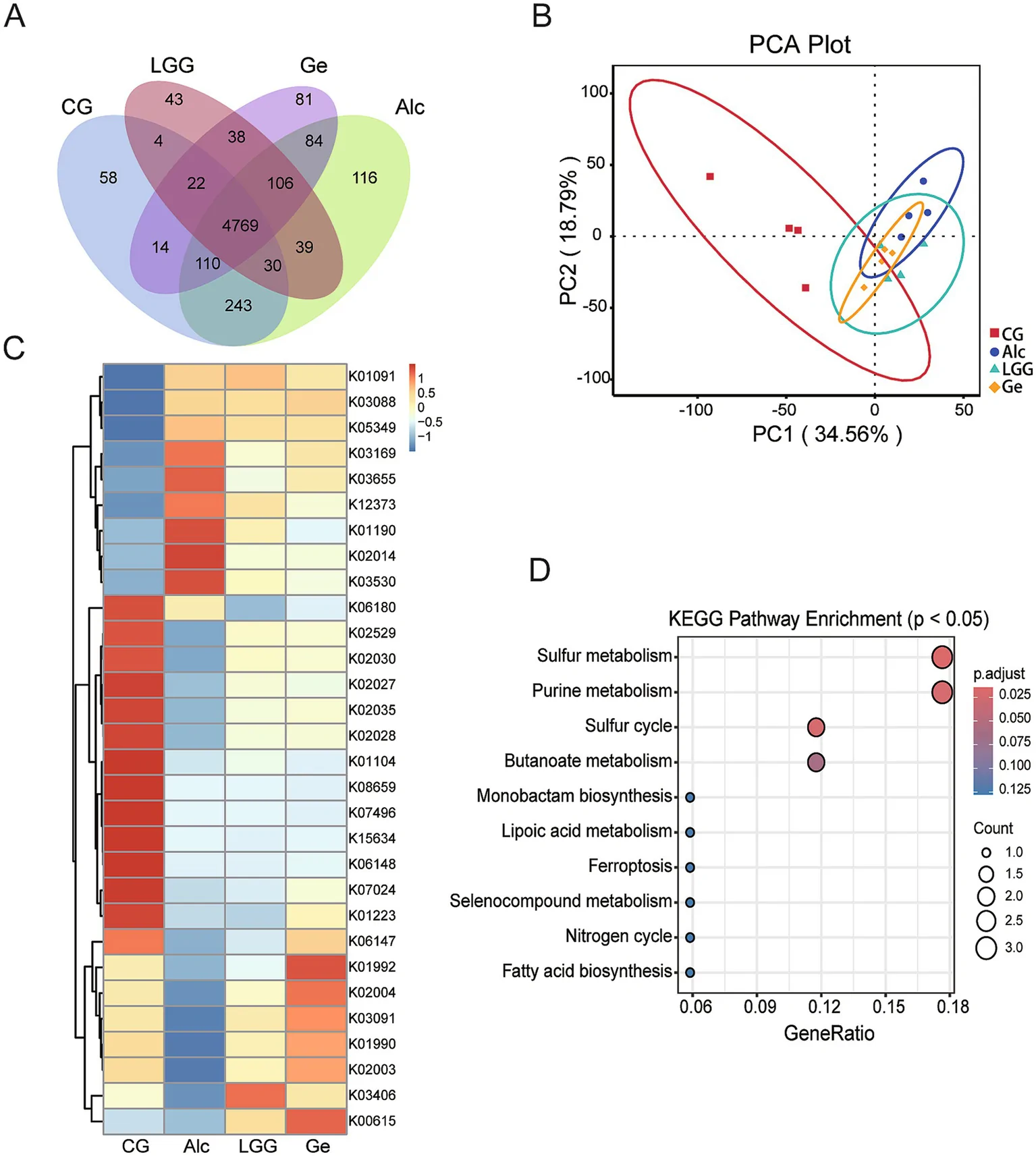
Functional prediction of gut microbiota in each group (*n* = 4 per group). **(A)** Venn diagram showing shared and unique KEGG Orthologs (KOs) among groups. **(B)** Principal component analysis (PCA) plot illustrating sample distribution based on predicted microbial functions. **(C)** Heatmap illustrating the relative abundance of predicted KEGG Orthologs (KOs) across different groups. **(D)** KEGG pathway enrichment analysis of differentially abundant functions among groups.

As shown in Figure 5C, several KOs, including K03169, K03655, K12373, K01190, K02014, and K03530, were notably elevated in the Alc group and declined to varying degrees in LGG and Ge group. In contrast, K06147, K01992, K02004, K03091, K01990, K02003, and K03406 showed reduced abundance in Alc group but were partially restored in the Lactobacillus-treated groups.

KEGG pathway enrichment analysis highlighted significant differences in pathways associated with sulfur metabolism, purine metabolism, the sulfur cycle, and butanoate metabolism (Figure 5D). These changes suggest that *Lactobacillus* may help restore intestinal barrier function and support anti-inflammatory and antioxidant activities. However, the exact molecular mechanisms driving these functional changes remain unclear and require further experimental investigation.

## Discussion

In this study, we examined changes in the gut microbiome of alcohol-fed rats and those treated with *Lactobacillus* using 16S rDNA sequencing. Chronic alcohol intake was found to disrupt the microbial community, resulting in intestinal dysbiosis, impaired barrier function, and systemic inflammation. In contrast, supplementation with *Lactobacillus* helped counteract these effects by rebalancing the gut microbiota, strengthening barrier integrity, and reducing inflammation.

Alcohol-related liver diseases remain a major global health concern and involve complex metabolic and inflammatory mechanisms, prompting the exploration of new therapeutic strategies (23). Alcohol consumption affected systemic and hepatic levels of fibroblast growth factor (FGF) 19 subfamily members—FGF19, FGF21, and FGF23—which are key endocrine regulators of metabolism, nutrient sensing, and energy balance (24). Among these, FGF21 is mainly produced by the liver and has widespread effects on multiple organs to maintain glucose and lipid balance (25). Consistent with previous studies showing that alcohol intake rapidly raises circulating FGF21 levels in humans (26), we observed a similar increase in alcohol-fed rats, indicating a conserved physiological response to ethanol exposure. Interestingly, the two *Lactobacillus* strains produced distinct effects. Supplementation with *L. gasseri* significantly lowered serum FGF21 compared with alcohol-fed rats, whereas *L. rhamnosus* further increased FGF21 levels. This suggests that different *Lactobacillus* species may have strain-specific influences on hepatic metabolic signaling. FGF21 acts both as a stress-inducible hepatokine and a protective metabolic regulator; its elevation is often linked to improved lipid metabolism, enhanced fatty acid oxidation, and reduced hepatic steatosis (25). Thus, the increase of FGF21 seen in LGG group may reflect an adaptive hepatoprotective response to alcohol-induced liver injury, potentially supporting lipid metabolism and recovery. Conversely, the decrease of FGF21 observed in Ge group might indicate reduced metabolic stress following microbial restoration, helping to normalize FGF21 expression. Alanine aminotransferase (ALT) and aspartate aminotransferase (AST) are enzymes mainly found in the cytoplasm and mitochondria of hepatocytes, they are released into the blood when liver cells are damaged. As such, the serum levels of ALT and AST are widely used as indicators of hepatic injury (27). In our study, chronic alcohol exposure led to a marked increase in both ALT and AST compared with control rats, reflecting significant liver damage. Notably, supplementation with *L. gasseri* significantly reduced these elevations, highlighting its protective effect on the liver. Although LGG group did not show a statistically significant decrease in ALT and AST levels, a downward trend was observed, suggesting it may partially mitigate alcohol-induced hepatic injury.

Excessive alcohol intake disrupts the balance of gut microbiota, promoting bacterial overgrowth, intestinal inflammation, and increased gut permeability (28). This compromise in mucosal integrity allows bacterial components, such as lipopolysaccharide (LPS), to enter the portal circulation. Once in the liver, LPS activates Toll-like receptor 4 (TLR4) on Kupffer cells and hepatic stellate cells, triggering the release of proinflammatory cytokines, including tumor necrosis factor-alpha (TNF-α) and interleukin-6 (IL-6) (29). This inflammatory response contributes to oxidative stress, hepatocyte apoptosis, and fibrogenesis—key features of alcoholic liver disease (ALD). In our study, chronic alcohol consumption elevated both serum LPS and hepatic TNF-α, confirming activation of gut–liver inflammatory signaling. Supplementation with *L. gasseri* significantly reduced these markers, suggesting improved gut barrier function and suppression of liver inflammation. *L. rhamnosus* also lowered hepatic TNF-α and modestly decreased serum LPS, indicating that both strains provide anti-inflammatory benefits, though to varying extents. These observations are consistent with previous reports showing that probiotics can protect against alcohol-induced liver injury by modulating the gut microbiota, enhancing tight junction proteins, and inhibiting LPS-triggered TLR4/NF-κB signaling (30). There is a two-way relationship between gut microbiota imbalance and alcohol use (13). On one hand, chronic alcohol consumption alters the composition and metabolic activity of intestinal microbes, causing dysbiosis, intestinal inflammation, and weakened gut barrier function. On the other hand, changes in the gut microbiota can affect alcohol-related behaviors and dependence through the “microbiome–gut–brain axis,” a communication network in which microbial metabolites interact with the enteric nervous system and central brain circuits involved in reward and stress regulation (12). Previous research in both animal models and patients with AUD has revealed diverse but generally consistent changes in the gut microbiota. For example, Wang et al. (31) reported increased levels of Clostridiales and Odoribacter in AUD mice, whereas Bull-Otterson et al. (32) observed decreases in Firmicutes and Bacteroidetes alongside increases in Proteobacteria and Actinobacteria in chronically alcohol-fed mice. Another study found that commensal microbes such as Lachnospiraceae, Prevotellaceae, and Ruminococcaceae were markedly reduced in AUD rats (33). Clinical studies support these findings. Bjørkhaug et al. (6) reported that patients with chronic alcohol intake showed a proinflammatory microbiota profile, with expansions of Proteobacteria and genera such as *Clostridium, Holdemania*, and *Sutterella*, along with a decline in *Faecalibacterium*, which is known for its anti-inflammatory and gut-protective roles. Similarly, Mutlu et al. (34) found reduced *Bacteroidetes* and *Lactobacillus*, accompanied by elevated *Proteobacteria* in alcohol-dependent patients. Leclercq et al. (35) further showed that *Lactobacillus* and *Bifidobacterium* were diminished in AUD patients but recovered following abstinence, highlighting the plasticity of the gut microbiota. Other studies have also reported decreases in mucin-degrading *Akkermansia muciniphila* and anti-inflammatory *Faecalibacterium prausnitzii*, indicating that alcohol disrupts microbial species critical for maintaining gut barrier function and immune balance (6, 36). In the present study, alcohol exposure increased the relative abundance of *Akkermansia*, *Alloprevotella*, *Bacteroides, Alistipes*, Rikenellaceae_RC9_gut_group, *Desulfovibrio*, and *Ruminococcus*, while reducing *Lactobacillus* and *Bifidobacterium*. Although there is some variation across studies, the loss of *Lactobacillus* and *Bifidobacterium* appears to be a consistent feature of alcohol-induced dysbiosis. These bacteria are important for butyrate production, which provides energy for colonocytes, strengthens epithelial tight junctions, and has anti-inflammatory effects (37). Their depletion may therefore weaken the gut barrier and facilitate translocation of endotoxins, contributing to systemic inflammation and liver injury. In our study, supplementation with *Lactobacillus* partially reversed these microbial changes. *Lactobacillus rhamnosus* reduced the relative abundance of *Alloprevotella, Alistipes,* and Rikenellaceae_RC9_gut_group, while *L. gasseri* lowered *Akkermansia, Alloprevotella,* and *Alistipes*. These shifts were accompanied by a restoration of microbial richness (α-diversity), suggesting that *Lactobacillus* treatment helped reestablish a more balanced gut ecosystem. It should be noted that the biological significance of these taxa is context-dependent. Although *Akkermansia muciniphila* has frequently been associated with improved intestinal barrier function and metabolic health (38), the direction and significance of changes in *Akkermansia* abundance may vary depending on the physiological and pathological context. Previous studies have reported both decreased and increased *Akkermansia* abundance following alcohol exposure, suggesting that its response to alcohol may be model- and context-dependent (9). In a mouse model of alcohol-associated liver disease, intestinal HIF-1α deficiency exacerbated alcohol-induced intestinal barrier dysfunction and liver injury, as evidenced by reduced intestinal tight-junction proteins, increased circulating LPS, and aggravated hepatic injury. Interestingly, increased *Akkermansia* abundance was observed concomitantly with these pathological changes, whereas *Lactobacillus* abundance was reduced (39). These findings indicate that an increase in *Akkermansia* may occur in the context of alcohol-associated intestinal barrier dysfunction, although they do not establish a causal role of *Akkermansia* in barrier disruption. Given the mucus-degrading characteristics of *Akkermansia*, one possible interpretation is that its expansion under conditions of alcohol-induced mucosal disturbance may reflect an adaptive or compensatory response to alterations in the intestinal mucus environment rather than a uniformly beneficial change. Therefore, the reduction of *Akkermansia* following *L. gasseri* treatment in our study should be interpreted as part of a broader remodeling of the gut microbial ecosystem rather than as evidence that *Akkermansia* is pathogenic.

Functional prediction using PICRUSt2 revealed notable differences in microbial metabolic pathways between groups. Specifically, KOs including K03169, K03655, K12373, K01190, K02014, and K03530, were significantly elevated in Alc group but showed moderate decreases in LGG and Ge group. Conversely, K06147, K01992, K02004, K03091, K01990, K02003, and K03406 decreased in Alc group and partly restored in LGG and Ge group. Some of the downregulated KOs in Alc group were linked to ATP-binding cassette (ABC) transporters, suggesting a reduced capacity for ATP-dependent transmembrane transport. ABC transporters are essential for importing nutrients and exporting xenobiotics and metabolic byproducts (40). Their decreased expression indicates impaired microbial nutrient uptake and detoxification, consistent with the metabolic stress and redox imbalance caused by chronic alcohol exposure (41). In contrast, the upregulated KOs may reflect compensatory activation of pathways related to genome stability, protein synthesis, and energy metabolism, as the microbiota adapts to ethanol-induced oxidative and inflammatory stress. KEGG pathway enrichment analysis further identified significant changes in sulfur metabolism, purine metabolism, sulfur cycling, and butanoate metabolism. These shifts suggest that *Lactobacillus* supplementation may support gut barrier repair and anti-inflammatory regulation by restoring key microbial metabolic networks. The enrichment of butanoate (butyrate) metabolism following *Lactobacillus* treatment is particularly noteworthy. Butyrate is a major short-chain fatty acid (SCFA) produced by gut microbes, which provides energy for colonocytes, strengthens epithelial tight junctions, and exerts anti-inflammatory effects through inhibition of the NF-κB signaling pathway (42, 43). The restoration of butyrate-producing capacity implies that *Lactobacillus* may help regenerate the intestinal barrier and reduce systemic inflammation through SCFA-mediated mechanisms.

In summary, our results showed that both *L. rhamnosus* and *L. gasseri* help to alleviate alcohol-induced dysbiosis, by restoring beneficial bacteria and reducing opportunistic pathogens. This modulation may promote intestinal barrier repair and immune regulation through complex interactions between microbial and host metabolism. These results suggest that targeting gut microbiota may be a potential strategy in the prevention and treatment of alcohol-related liver injury. However, our study has several limitations. First, the sample size was relatively small, and only four biological replicates per group were included in the 16S rRNA gene sequencing analysis. The selection of samples was not based on a predefined randomization procedure, and therefore potential selection bias cannot be completely excluded. However, samples were not selected according to alcohol consumption, behavioral performance, or other experimental outcomes, and the same sampling approach was applied across all experimental groups. Future studies with a larger number of biological replicates and prospective randomization of samples are warranted to validate these microbiome findings. Second, only male rats were used in this study. Although the use of male rats reduces biological variability associated with sex hormones and the estrous cycle, it may limit the generalizability of our findings. Estrogen has been reported to exert protective effects on mitochondrial function and hepatic metabolism by attenuating oxidative stress and improving cellular energy homeostasis, which may influence susceptibility to alcohol-associated liver injury (44). Future studies including female animals will be necessary to determine potential sex-specific responses to probiotic interventions. Third, while 16S rDNA sequencing provided an overview of gut microbial changes, it does not capture species-level resolution or functional gene activity. Future studies incorporating metagenomic or metabolomic analyses would help clarify the underlying mechanisms. Finally, our focus was primarily on microbial and biochemical outcomes, without examining neuroendocrine effects associated with alcohol dependence. More work need to be done to better understand how probiotic interventions influence alcohol-related systemic effects.

## Conclusion

In conclusion, our study demonstrates that chronic alcohol consumption disrupts gut microbial balance, impairs intestinal barrier function, and contributes to systemic inflammation and liver injury. Supplementation with *L. rhamnosus* and *L. gasseri* alleviated these effects by restoring beneficial bacteria and reducing hepatic and systemic inflammatory responses. These protective effects were linked to changes in key metabolic pathways, including ABC transporters, sulfur and purine metabolism, and butanoate metabolism, suggesting that *Lactobacillus* acts through coordinated regulation of microbial and host metabolism. Overall, our findings emphasize the importance of the gut microbiota in alcohol-related liver injury and support targeted probiotic intervention as a potential strategy for prevention and treatment of alcohol-induced liver disorders.

## Data Availability

The raw sequence data reported in this paper have been deposited in the Genome Sequence Archive (Genomics, Proteomics & Bioinformatics 2025) in National Genomics Data Center (Nucleic Acids Res 2025), China National Center for Bioinformation / Beijing Institute of Genomics, Chinese Academy of Sciences (GSA: CRA050297) that are publicly accessible at https://ngdc.cncb.ac.cn/gsa.

## Supplementary material

SUPPLEMENTARY FIGURE S1 Results of *t*-test analyses comparing microbial composition between groups at the phylum **(A)** and genus **(B)** levels (*n* = 4 per group).
